# Does Atrial Fibrillation at Diagnosis Change Prognosis in Patients with Aortic Stenosis?

**DOI:** 10.3390/jcm13133917

**Published:** 2024-07-04

**Authors:** Olga Petrovic, Stasa Vidanovic, Ivana Jovanovic, Ivana Paunovic, Ivana Rakocevic, Dejan Milasinovic, Milorad Tesic, Nikola Boskovic, Djordje Dukic, Marina Ostojic, Jelena Vratonjic, Aleksandra Mladenovic, Danijela Trifunovic-Zamaklar

**Affiliations:** 1 Department of Cardiology, University Clinical Center of Serbia, 11000 Belgrade, Serbia; ivana170679@gmail.com (I.J.); inapaunovic@gmail.com (I.P.); ivanarakocevic11@gmail.com (I.R.); milasin.d18@gmail.com (D.M.); misa.tesic@gmail.com (M.T.); belkan87@gmail.com (N.B.); djoleczv1994@gmail.com (D.D.); drmarinaostojic@gmail.com (M.O.); j_dudic@hotmail.com (J.V.); danijelatrif@gmail.com (D.T.-Z.); 2 Faculty of Medicine, University of Belgrade, 11000 Belgrade, Serbia; stasavidanovic99@gmail.com (S.V.); sandraaamladenovic@gmail.com (A.M.)

**Keywords:** aortic stenosis, atrial fibrillation, mortality

## Abstract

**Background:** Aortic stenosis (AS) is a common valve disease and atrial fibrillation (AF) is the most common cardiac arrhythmia, frequently associated with AS. This study aimed to evaluate the impact of AF on mortality in patients with moderate and severe AS. **Methods:** We retrospectively analyzed 1070 consecutive moderate and severe AS patients (57% were male, age was 69 ± 10, severe AS 22.5%), who underwent transthoracic echocardiography from March 2018 to November 2021. AS severity was defined by specific threshold values with severe AS being defined by a peak velocity > 4 m/s, an MPG > 40 mmHg, and an AVA < 1 cm^2^ and moderated by a peak velocity of 3–4 m/s, an MPG 20–40 mmHg and an AVA 1–1.5 cm. Patients with AF were defined as those having a history of AF when AS was found on the index echocardiography. The follow-up assessment in December 2023 ascertained vital status and data on aortic valve replacement (AVR). **Results:** 790 (73.8%) patients were with sinus rhythm (SR) and 280 (26.2%) patients with AF. Mortality was higher in patients with AF than in those with SR (46% vs. 36.2% HR 1.424, 95% CI 1.121–1.809, *p* = 0.004). After adjusting for clinical confounders, mortality risk in AF relative to SR remained significant (HR 1.284, 95% CI 1.03–1.643, *p* = 0.047). Patients with AF demonstrated high mortality risk in the moderate aortic stenosis stratum (HR 1.376, 95% CI 1.059–1.788, *p* = 0.017), with even greater risk in the severe AS stratum (HR 1.644, 95% CI 1.038–2.603, *p* = 0.034) with significant interaction (*p* = 0.007). In patients with AF AVR demonstrated a protective effect on survival (HR 0.365, 95% CI 0.202–0.627, *p* < 0.001), but to a lesser degree than in patients with sinus rhythm (HR 0.376, 95% CI 0.250–0.561, *p* < 0.001) without significant interaction (*p* = 0.278). In patients with AF mortality risk was high in the conservative treatment stratum (HR 1.361, 95% CI 1.066–1.739, *p* = 0.014), in the AVR stratum mortality risk was higher but did not reach statistical significance (HR 1.823, 95% CI 0.973–3.414, *p* = 0.061). However, when corrected for echocardiographic variables strongly correlated with AF, AF was no longer independently associated with all-cause mortality. (HR 0.97 95% CI 0.709–1.323, *p* = 0.84). **Conclusions:** Patients with moderate and severe AS and AF have worse prognosis than patients with SR which can be explained by cardiac damage. AVR improves survival in patients with AF and with SR.

## 1. Introduction

The most common valvular disease, Aortic Stenosis (AS) is a cause of significant mortality and morbidity, yet no effective preventive treatment exists, despite well-established risk factors and pathophysiological mechanisms [1]. Worldwide, the most common long-term heart rhythm disorder is atrial fibrillation (AF) [2].

AF and aortic valve stenosis share many common risk factors, including age and hypertension, and aortic valve stenosis alone is associated with a higher rate of AF. A higher incidence of AS and AF is expected since the world population is becoming older, and aging is a significant risk factor for both conditions [3].

The timing of intervention is crucial in the treatment of aortic stenosis. Aortic valve replacement remains the only treatment proven to reduce the rates of mortality and morbidity in severe aortic stenosis [4]. According to current guidelines, replacement of the aortic valve (AVR) should be considered for patients who have severe AS accompanied by symptoms or evidence of left ventricular dysfunction (left ventricular ejection fraction LVEF < 50%) [5].

Moderate AS is not a benign condition. The risk of cardiac and noncardiac mortality is substantially increased as a result of this disease. Mortality rates in moderate aortic stenosis are similar to rates observed in patients with severe AS [6]. The progression of the disease to a severe stage can also occur much faster than previously expected [7]. Extra valvular cardiac damage, especially the extent of myocardial hypertrophy is not always correlated with the severity of aortic stenosis [8]. AF is more frequent in patients with AS than in patients with no AS because of pressure overload resulting in left ventricular (LV) hypertrophy, and left atrial (LA) enlargement, and can be a negative prognostic sign of deterioration of AS [9].

Although current guidelines do not recommend AVR in moderate AS; however, these patients might also have symptoms that are not easily explained by any other cause. Furthermore, atrial fibrillation can cause dizziness, fatigue, and shortness of breath.

In this study, we aimed to determine if the presence of atrial fibrillation was associated with a higher mortality rate in patients who had moderate and severe aortic stenosis.

## 2. Materials and Methods

### 2.1. Study Population

We retrospectively analyzed 1070 patients with moderate and severe AS, who underwent transthoracic echocardiography (TTE) from November 2018 to March 2021. at the University Clinical Center of Serbia.

### 2.2. Data Collection and Study Definitions

The aortic valve peak velocity (PV), mean pressure gradient across the aortic valve (MPG), and aortic valve area (AVA) using the Doppler continuity equation, and degree of mitral regurgitation (MR) were recorded. The severity of stenosis was defined according to the 2021 European Society of Cardiology guidelines as follows: moderate AS - PV ≥ 3–4 m/s, MPG ≥ 20–40 mmHg, and AVA 1–1.5 cm^2^; severe AS - PV > 4 m/s, MPG > 40 mmHg, and an AVA < 1 cm^2^ [5,10,11]. Mitral regurgitation (MR) grade < 2 is considered mild and grade ≥ 2 is considered moderate to severe. For patients with sinus rhythm (SR), three consecutive cardiac cycles were averaged for all measurements. For patients with AF, at least five consecutive cardiac cycles were averaged. Pulmonary hypertension was diagnosed by indirect right ventricular systolic pressure (RVSP) estimation using tricuspid regurgitation (TR) jet velocity yielding a peak pressure of >40 mmHg. Simpson’s biplane method was used for left ventricular ejection fraction (EF) assessment (EF ≤ 50% was considered reduced left ventricular systolic function).

AF is defined as an irregular atrial rhythm with the absence of consistent P waves [12]. Using the database, we analyzed patients for the presence or absence of AF. A patient with AF was defined as one with a history of AF or documented by 12-lead ECG at the time of AS diagnosis. According to electrocardiographic findings, patients were divided into two groups: SR and AF.

### 2.3. Study Outcomes

The follow-up assessment in December 2023 ascertained vital status and data on AVR. Medical records were reviewed retrospectively, and telephone calls were made to patients or their next of kin to obtain follow-up information. The impact of AVR on survival was analyzed as a time-dependent covariate.

According to the data obtained from previous sources, we determined mortality in moderate and severe AS patients and the frequency of AF. We evaluated whether AF is an independent risk factor for all-cause mortality and how it is affected by AVR and degree of stenosis.

### 2.4. Statistical Analysis

Continuous variables are presented as means ± standard deviation (SD), while categorical variables are presented as frequencies and percentages. The normality of distribution for numerical characteristics was tested using the Kolmogorov–Smirnov test. Differences between groups were analyzed by the Student’s *t*-test for continuous variables and Pearson’s chi-squared test for categorical variables. A Kaplan–Meier mortality curve was constructed, and the difference was analyzed using a two-sided Log-rank test. The Cox regression model was used to determine independent predictors of mortality. In the selection of variables for multivariate analysis, the first univariate regression analysis was performed and only variables that showed statistical significance were included in the multivariate analysis. We analyzed and compared whether there is statistical significance between the stratum of moderate and the stratum of severe AS, as well as the stratum of conservative treatment and AVR stratum. Hazard ratios (HRs) for mortality are presented with their 95% confidence intervals (CIs). All tests were 2-sided. A value of *p* < 0.05 was considered statistically significant, and a value of *p* < 0.01 is highly statistically significant. Data were analyzed using SPSS statistical software version 23.

## 3. Results

### 3.1. Baseline Characteristics

This retrospective study included 1070 patients (829 (77.5%) patients with moderate AS and 241 (22.5%) patients with severe AS, 608 (56.8%) of them male. The mean age was 69 ± 10 years. On the echocardiography assessment, the average value of a PV was 3.9 ± 1.89 m/s, an MPG was 37 ± 15.42 mmHg, and an AVA was 0.82 ± 0.27 cm^2^.

In Table 1, we present a summary of the baseline characteristics of the study population based on the cardiac rhythm. Compared with 790 (73.8%) patients with SR, 280 (26.2%) patients with AF were older (*p* < 0.001) and more likely to have diabetes mellitus (*p* = 0.02). The incidence of comorbidities, such as arterial hypertension, stroke, myocardial infarction, pulmonary comorbidities, and chronic kidney disease, was comparable between the two groups.

In Table 2, we present the baseline echocardiographic parameters of the study population based on their cardiac rhythm. Patients with AF had larger left atrial diameter (*p* < 0.001), lower left ventricular EF (*p* < 0.001), greater degree of mitral regurgitation (*p* < 0.001) and greater estimated RVSP (*p* < 0.001).

The percentages of AF were similar between patients with moderate AS 220/829 (26.53%) and severe AS 60/241 (24.89%), *p* = 0.67

Table 3 describes a subgroup analysis considering the presence of atrial fibrillation. A subgroup analysis was conducted to determine whether the presence of atrial fibrillation is associated with aortic stenosis severity or has affected the treatment approach. There was no statistically significant difference.

### 3.2. Follow-Up

At the time of data collection transcatheter aortic valve replacement was not available at our center. The only option for aortic valve replacement was surgical.

**Figure 1:**
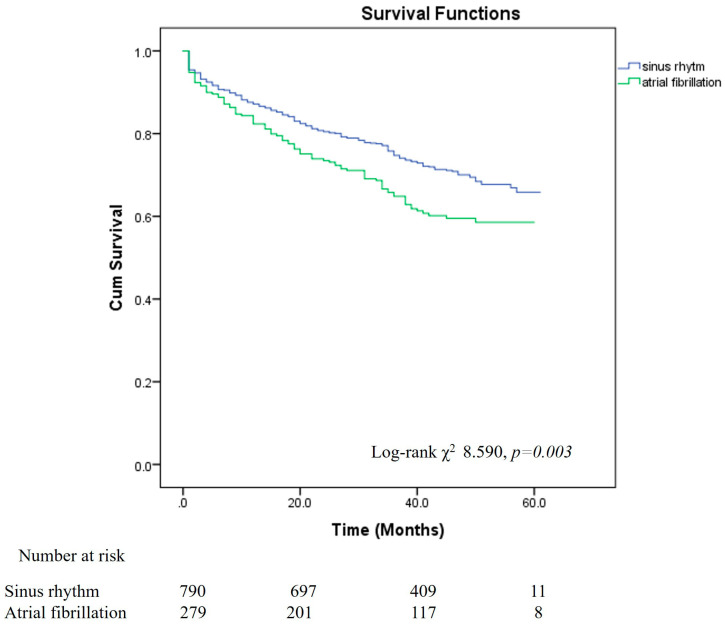
Kaplan–Meier curve. Overall survival rate of patients with moderate and severe AS and AF (1) compared to patients with moderate and severe AS and SR (0). The curves were compared using the log-rank test (*p* = 0.003).

At the time of follow-up, there were 655 (61.21%) patients alive and 415 (38.78%) deceased. Both patients with moderate and severe AS patients have an increased risk of death with similar mortality rates (severe AS 40% (332/829), moderate AS 34.4% (83/241), *p* = 0.133). AF group had significantly higher mortality 46% (129/280) than the sinus rhythm group 36.2% (286/790),

Survival curves were plotted by the Kaplan–Meier method and compared using the log-rank test (*p* = 0.003). (Figure 1) During a median 41-month follow-up the risk of death (hazard ratio) of moderate to severe AS patients with AF as compared to those with SR was 1.424 (95% CI, 1.121–1.809; *p* = 0.004). The average overall survival was 46.8 months (95% confidence interval [CI], 45.5–48.2), also in patients with AF the average survival was 49.2 months (95% CI, 40.12–45.71), and in patients without AF was 47.9 months (95% CI, 46.43–49.53).

**Figure 2:**
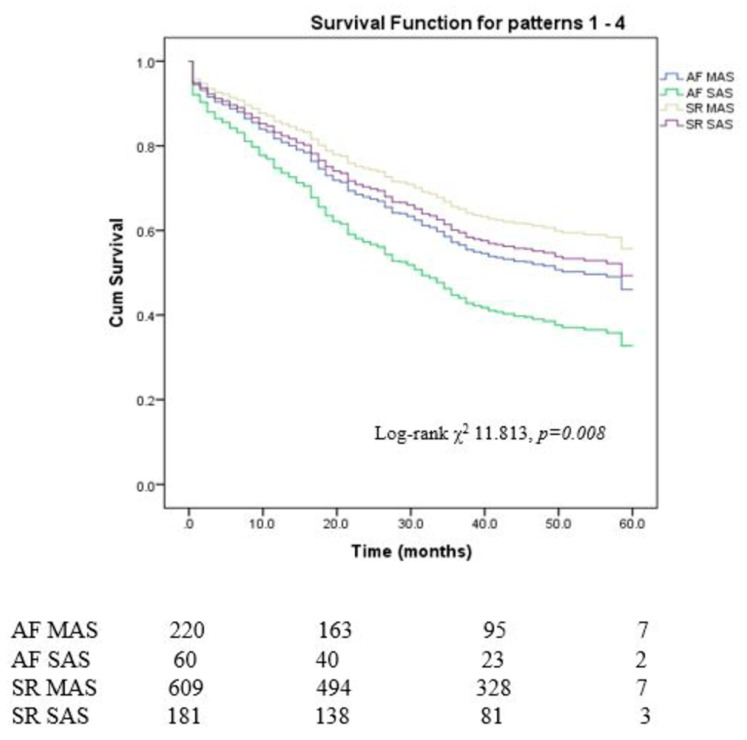
Kaplan–Meier estimates of cumulative event-free survival of the study population stratified by aortic stenosis severity and presence of atrial fibrillation The curves were compared using the log-rank test (*p* = 0.008). Abbreviations: AF—Atrial fibrillation, MAS—moderate aortic stenosis, SAS—severe aortic stenosis.

Study participants were separated based on the presence of severe AS and atrial fibrillation. Figure 2 displays the Kaplan–Meier curves of cumulative event-free survival for the various groups.

Among patients with moderate AS, patients with AF had a lower average overall survival than patients in SR (40 months vs. 44 months, respectively; *p* = 0.022). Amongst patients with severe AS, an additional effect of AF on average survival was observed (41.7 months for SR vs. 33.2 months for AF, *p* = 0.05). Patients with severe AS in SR had similar average survival compared to the patients with moderate AS in AF (41.7 months vs. 44 months, *p* = 0.557) 

Patients with AF demonstrated high mortality risk in the moderate aortic stenosis stratum (HR 1.376, 95% CI 1.059–1.788, *p* = 0.017), in the severe AS stratum the risk of death was higher (HR 1.644, 95% CI 1.038–2.603, *p* = 0.034) with significant interaction (*p* = 0.007) indicating a differential effect of arrhythmias whether patients were in AF or SR. In patients with AF AVR demonstrated a protective effect on survival (HR 0.365, 95% CI 0.202–0.627, *p* < 0.001), but to a lesser degree than in pts with sinus rhythm (HR 0.376, 95% CI 0.250–0.561, *p* < 0.001) without significant interaction (*p* = 0.278). In patients with AF mortality risk was high in the conservative treatment stratum (HR 1.424, 95% CI 1.068–1.899, *p* = 0.016), in the AVR stratum mortality risk was higher but did not reach statistical significance (HR 1.789, 95% CI 0.959–3.338, *p* = 0.067)

**Figure 3:**
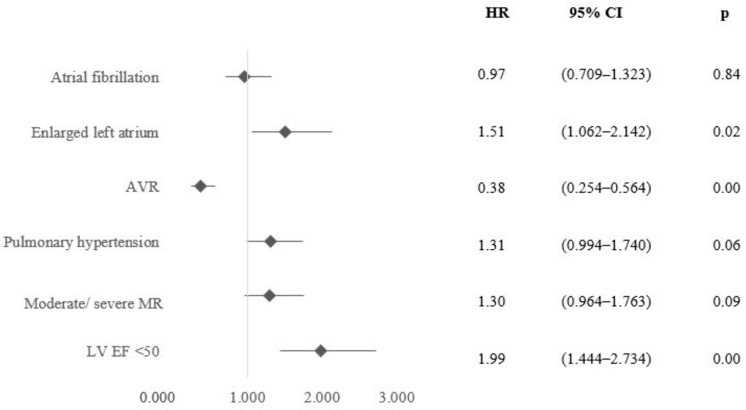
Multivariable predictors of 5-year mortality (echocardiography, heart rhythm, and time-dependent AVR). Abbreviations: AVR—aortic valve replacement, MR—mitral regurgitation, LV EF—left ventricular ejection fraction.

Factors associated with 5-year mortality are presented in Figure 3. Enlarged left atrium (*p* = 0.02) and EF < 50% (*p* < 0.001), are independent mortality predictors in patients with moderate to severe AS, with AVR acting protectively (*p* < 0.001).

## 4. Discussion

The purpose of this study was to determine whether AF was associated with poorer survival in patients with moderate and severe AS. The main findings are (i) AF is prevalent in a cohort of patients with moderate and severe AS. (ii) After adjusting for clinical factors, mortality was higher in AF relative to SR. (iii) Patients in SR and severe AS had similar average survival as patients in AF and moderate AS. (iv) However, when corrected for echocardiographic variables strongly correlated with AF, AF was no longer independently associated with all-cause mortality. (v) In patients with AF AVR demonstrated a protective effect on survival, but to a lesser degree than in patients with SR.

AS is one of the most common valvular diseases, the diagnosis of which is increasing, secondary to the advances in echocardiography, but there are still no known ways to prevent it. Moderate and severe grades of AS share an ominous prognosis if left untreated. In both moderate and severe AS, mortality is high and not significantly different [13].

The prognosis often depends on associated conditions. Arterial hypertension (AH) contributes to left ventricular remodeling and worsens the prognosis of these patients. AH also interferes with the echocardiographic assessment of transaortic gradients. The severity of AS may be underestimated in the presence of coexisting AH. The prevalence of AH in AS is high. Up to 70% of patients with AS have coexisting AH [14]. In our study, AH was present in 50% of patients.

The incidence of AH and most comorbidities was comparable between the two groups. Patients with AF were older and more likely to have diabetes mellitus (DM) which is not surprising considering that the causal relation between DM and AF is recognized. DM is an independent risk factor for AF by promoting left atrial remodeling and arrhythmogenesis [15,16]. Comparable to previous reports, our study demonstrated similar all-cause mortality risks in patients with moderate and severe aortic stenosis [6,13].

The frequency of pre-operative AF in patients with AS referred for AVR is estimated to be between 16 and 35%. Differences in AF definition and methods of detection likely lead to this wide-range incidence. In our study, AF was present in 26.53% of patients with moderate AS and 24.89% of patients with severe AS which is aligned with previous reports [17,18,19].

In the general population, AF leads to a 1.5 to 1.9-fold increase in the risk of death [9]. Multiple studies have shown that AF is an independent predictor of mortality in AS patients irrespective of stenosis severity and treatment modality and that AVR compared to conservative treatment, reduces mortality [18,20,21,22]. Kubala et al. showed in 1838 patients that in severe AS (32% with AF), AF is a strong predictor of mortality even in asymptomatic patients, but in these patients, AVR was associated with lower mortality compared with conservative treatment [18]. In 508 patients with asymptomatic or minimally symptomatic moderate AS and preserved EF (33.7% with AF), Delesalle et al. showed that AF was independently associated with all-cause mortality and that AVR was associated with better survival [22]. After adjustment for known associated factors, our study also showed that patients with atrial fibrillation had a higher rate of all-cause mortality than those without atrial fibrillation.

Not all studies showed that AVR has a protective effect on the survival of patients with moderate to severe AS. Matsuda et al. analyzed 3815 patients with severe AS enrolled in the CURRENT AS registry and reported that the magnitude of excess adjusted risk of AF for the primary outcome measure was greater in the initial AVR stratum than in the conservative stratum [20]. Our study demonstrated that in patients with AF, AVR had a protective effect on survival but to a lesser degree than in pts with sinus rhythm.

Some studies did not prove an independent association between AF and all-cause mortality in patients with AS. Zhang et al. in their study including 1867 patients with AS showed nearly the same results regarding the poor prognosis of patients with AF and severe AS, and pointed out the importance of timely AVR, especially due to the frequent attribution of symptoms of AS to AF, but AF was not a predictor of mortality independent of variables strongly correlated [23]. Leanens et al. evaluated 2849 patients with severe AS and the prognostic impact of AF in patients with AS while correcting for LV diastolic function. AF was not independently associated with outcomes in patients with significant AS [24]. Our results also imply that AF is strong but not an independent risk factor in patients with AS. A possible explanation for this finding is that when present, AF implies that significant structural heart remodeling had already happened AS provokes left ventricular hypertrophy and left atrial enlargement which are substrates for AF occurrence and perpetuation. The presence of AF provokes symptoms in AS by reducing cardiac output and increasing filling pressures. The prognostic impact of AF seems to be the same in moderate and severe grades of AS raising concern that moderate AS can be as fatal as severe AS.

Murphy et al. in patients with moderate AS, showed that AF, MR, EF, and pulmonary hypertension affect survival [19]. Similar results were obtained in this study, it was observed that with the increase in the dimensions of the left atrium, the larger MR, lower EF, and AF increase the negative impact on patient survival.

## 5. Limitations

Our study has some limitations. This is a retrospective single-center study. AF diagnosis was based on the presence of AF at the time of the echocardiography exam from previous medical records and a 12-lead ECG at the time of the AS diagnosis. A 24-h ambulatory ECG monitoring was not routinely performed; therefore, real AF burden was unclear. Atrial fibrillation often occurs after cardiac surgery and is associated with increased mortality. Patients with postoperative atrial fibrillation were not identified.

## 6. Conclusions

Patients with moderate and severe AS and AF have worse prognosis than patients in SR which can be explained by cardiac damage. AVR improves survival both in patients in AF and SR. Aortic stenosis affects not only the valve but also the myocardium, and a significant proportion of patients with moderate stenosis will also suffer from left ventricular dysfunction. Currently, there are no recommendations regarding additional echocardiographic features that can be used to determine the timing of intervention in patients with aortic stenosis other than LVEF and aortic stenosis severity. The study’s most important clinical implication is the need to incorporate more imaging parameters in decision-making. It is necessary to conduct new randomized clinical trials to assess the hypothesis that imaging-driven care coupled with upstream intervention will result in better long-term clinical outcomes in aortic valve disease. Some patients with moderate aortic stenosis may benefit from earlier intervention.

## Figures and Tables

**Table 1:** Clinical characteristics according to cardiac rhythm.

	Atrial Fibrillation	Sinus Rhythm	*p* Value
Male (%)	166 (59.28)	442 (55.95)	0.369
Age	71.52 ± 10.47	68.97 ± 10.38	<0.001
Myocardial infarction (%)	26 (9.3)	63 (7.9)	0.725
Hipertension (%)	155 (55.3)	392 (46.6)	0,1
Diabetes melitus (%)	69 (24.6)	143 (18.1)	0.02
Hyperlipidemia (%)	43 (15.3)	154 (19.5)	0.148
Pulmonary comorbidities (%)	21 (7.5)	48 (6)	0.489
Nephrological comorbidities (%)	26 (9.3)	45 (5.7)	0.053
Stroke (%)	16 (5.7)	34 (4.3)	0.327
AVR	76 (7.1)	194 (18)	0.337

Abbreviations: AVR—Aortic valve replacement.

**Table 2:** Echocardiographic parameters according to cardiac rhythm.

	Atrial Fibrillation	Sinus Rhythm	*p* Value
LAD (cm)	4.77 ± 0.86	4.23 ± 0.59	<0.001
LVEF (%)	56.15 ± 13.23	61.20 ± 11.13	<0.001
PV (m/s)	3.9 (1.8–58)	3.97 ± 0.7	0.156
MPG (mmHg)	36 (7–93)	37 (2.3–111)	0.982
AVA (cm^2^)	0.79 (0.25–1.8)	0.8 (0.12–2.1)	0.087
MR grade	2 (1–3.5)	1 (1–3.5)	<0.001
RVSP (mmHg)	43.19 ± 12.62	39.33 ± 11.5	<0.001

Abbreviations: LAD—Left atrial diameter, LVEF—Left ventricular ejection fraction, PV—Peak velocity, MPG—Mean pressure gradient, AVA—Aortic valve area, MR—Mitral regurgitation, RVSP—Right ventricular systolic pressure.

**Table 3:** Subgroup analysis according to cardiac rhythm.

		Atrial Fibrillation	Sinus Rhythm	*p*-Value
Type of stenosis	Moderate AS	220 (26.53)	609 (73.46)	0.67
	Severe AS	60 (24.89)	181 (75.1)	
Treatment strategy	Conservative	194 (24.6)	596 (75.4)	0.218
	AVR	76 (27.1)	60 (72.9)	

Abbreviations: AS—Aortic stenosis, AVR—Aortic valve replacement.

## Data Availability

The authors declare that the gathered data included and used for the analysis outline are available in the manuscript. Further datasets are available upon reasonable request from the corresponding author.

## References

[1] Nkomo V.T., Gardin J.M., Skelton T.N., Gottdiener J.S., Scott C.G., Enriquez-Sarano M. (2006). Burden of valvular heart diseases: A population-based study. Lancet.

[2] Wyndham C.R. (2000). Atrial fibrillation: The most common arrhythmia. Tex. Heart Inst. J..

[3] Zebhi B., Lazkani M., Bark D. (2021). Calcific Aortic Stenosis-A Review on Acquired Mechanisms of the Disease and Treatments. Front. Cardiovasc. Med..

[4] Otto C.M., Prendergast B. (2014). Aortic-Valve Stenosis—From Patients at Risk to Severe Valve Obstruction. N. Engl. J. Med..

[5] Vahanian A., Beyersdorf F., Praz F., Milojevic M., Baldus S., Bauersachs J., Capodanno D., Conradi L., De Bonis M., De Paulis R. (2022). 2021 ESC/EACTS Guidelines for the management of valvular heart disease. Eur. Heart J..

[6] Strange G., Stewart S., Celermajer D., Prior D., Scalia G.M., Marwick T., Ilton M., Joseph M., Codde J., Playford D. (2019). Poor Long-Term Survival in Patients With Moderate Aortic Stenosis. J. Am. Coll. Cardiol..

[7] Palta S., Pai A.M., Gill K.S., Pai R.G. (2000). New Insights Into the Progression of Aortic Stenosis. Circulation.

[8] Carabello B.A. (2013). How Does the Heart Respond to Aortic Stenosis. Circ. Cardiovasc. Imaging.

[9] Benjamin E.J., Wolf P.A., D’Agostino R.B., Silbershatz H., Kannel W.B., Levy D. (1998). Impact of atrial fibrillation on the risk of death: The Framingham Heart Study. Circulation.

[10] Baumgartner H., Hung J., Bermejo J., Chambers J.B., Edvardsen T., Goldstein S., Lancellotti P., LeFevre M., Miller F., Otto C.M. (2016). Recommendations on the echocardiographic assessment of aortic valve stenosis: A focused update from the European Association of Cardiovascular Imaging and the American Society of Echocardiography. Eur. Heart J.—Cardiovasc. Imaging.

[11] Lang R.M., Badano L.P., Mor-Avi V., Afilalo J., Armstrong A., Ernande L., Flachskampf F.A., Foster E., Goldstein S.A., Kuznetsova T. (2015). Recommendations for Cardiac Chamber Quantification by Echocardiography in Adults: An Update from the American Society of Echocardiography and the European Association of Cardiovascular Imaging. Eur. Heart J.—Cardiovasc. Imaging.

[12] Hindricks G., Potpara T., Dagres N., Arbelo E., Bax J.J., Blomström-Lundqvist C., Boriani G., Castella M., Dan G.-A., Dilaveris P.E. (2020). 2020 ESC Guidelines for the diagnosis and management of atrial fibrillation developed in collaboration with the European Association for Cardio-Thoracic Surgery (EACTS): The Task Force for the diagnosis and management of atrial fibrillation of the European Society of Cardiology (ESC) Developed with the special contribution of the European Heart Rhythm Association (EHRA) of the ESC. Eur. Heart J..

[13] Généreux P., Sharma Rahul P., Cubeddu Robert J., Aaron L., Abdelfattah Omar M., Koulogiannis Konstantinos P., Marcoff L., Naguib M., Kapadia Samir R., Makkar Rajendra R. (2023). The Mortality Burden of Untreated Aortic Stenosis. J. Am. Coll. Cardiol..

[14] Linhartová K., Filipovský J., Cerbák R., Sterbáková G., Hanisová I., Beránek V. (2007). Severe aortic stenosis and its association with hypertension: Analysis of clinical and echocardiographic parameters. Blood Press..

[15] Wang A., Green J.B., Halperin J.L., Piccini J.P. (2019). Atrial Fibrillation and Diabetes Mellitus: JACC Review Topic of the Week. J. Am. Coll. Cardiol..

[16] Ugowe F.E., Jackson L.R., Thomas K.L. (2019). Atrial Fibrillation and Diabetes Mellitus. Circ. Arrhythmia Electrophysiol..

[17] Stortecky S., Buellesfeld L., Wenaweser P., Heg D., Pilgrim T., Khattab A.A., Gloekler S., Huber C., Nietlispach F., Meier B. (2013). Atrial Fibrillation and Aortic Stenosis. Circ. Cardiovasc. Interv..

[18] Kubala M., Bohbot Y., Rusinaru D., Maréchaux S., Diouf M., Tribouilloy C. (2023). Atrial fibrillation in severe aortic stenosis: Prognostic value and results of aortic valve replacement. J. Thorac. Cardiovasc. Surg..

[19] Murphy K.R., Khan O.A., Rassa A.C., Elman M.R., Chadderdon S.M., Song H.K., Golwala H., Cigarroa J.E., Zahr F.E. (2019). Clinical and Echocardiographic Predictors of Outcomes in Patients With Moderate (Mean Transvalvular Gradient 20 to 40 mm Hg) Aortic Stenosis. Am. J. Cardiol..

[20] Matsuda S., Kato T., Morimoto T., Taniguchi T., Minamino-Muta E., Matsuda M., Shiomi H., Ando K., Shirai S., Kanamori N. (2023). Atrial fibrillation in patients with severe aortic stenosis. J. Cardiol..

[21] Tarantini G., Mojoli M., Windecker S., Wendler O., Lefèvre T., Saia F., Walther T., Rubino P., Bartorelli A.L., Napodano M. (2016). Prevalence and Impact of Atrial Fibrillation in Patients With Severe Aortic Stenosis Undergoing Transcatheter Aortic Valve Replacement: An Analysis From the SOURCE XT Prospective Multicenter Registry. JACC Cardiovasc. Interv..

[22] Delesalle G., Bohbot Y., Rusinaru D., Delpierre Q., Maréchaux S., Tribouilloy C. (2019). Characteristics and Prognosis of Patients With Moderate Aortic Stenosis and Preserved Left Ventricular Ejection Fraction. J. Am. Heart Assoc..

[23] Zhang H., El-Am E.A., Thaden J.J., Pislaru S.V., Scott C.G., Krittanawong C., Chahal A.A., Breen T.J., Eleid M.F., Melduni R.M. (2020). Atrial fibrillation is not an independent predictor of outcome in patients with aortic stenosis. Heart.

[24] Laenens D., Stassen J., Galloo X., Ewe S.H., Singh G.K., Ammanullah M.R., Hirasawa K., Sia C.-H., Butcher S.C., Chew N.W.S. (2023). The impact of atrial fibrillation on prognosis in aortic stenosis. Eur. Heart J.—Qual. Care Clin. Outcomes.

